# The Influence of Missing Data Handling Methods on CES-D Analysis in the China Health and Retirement Longitudinal Study (CHARLS)

**DOI:** 10.21203/rs.3.rs-9662270/v1

**Published:** 2026-06-05

**Authors:** Xiaoyu Mu, Hao Ran Ma, Henry S Lynn

**Affiliations:** Xinjiang Medical University; Xinjiang Medical University; Xinjiang Medical University

**Keywords:** CES-D-10, CHARLS, Missing data, Multiple imputation

## Abstract

Most evaluations of missing data analyses rely on literature reviews or comparisons of hypothesized methods applied to a given dataset, yet the real-world impact of different strategies remains unknown. Using the widely analyzed CHARLS dataset, we reviewed missing data handling methods in 219 studies using the CES-D-10, and compared the three most prevalent procedures: complete-case analysis (CCA), impute zero (IZ), and multiple imputation (MI). Although CES-D-10 items were not missing completely at random, 90% of publications used CCA, resulting in lower mean CES-D-10 scores but higher depression prevalence, especially among adults aged 75 and older. The IZ CES-D-10 score constructed in the harmonized CHARLS dataset systematically underestimated both prevalence and mean scores, distorting age trajectories and attenuating sex differences in later life. Many CHARLS-based depression studies are therefore likely affected by systematic bias due to suboptimal treatment of missing CES-D-10 items, particularly those studies using the harmonized dataset. Moreover, because the IZ approach has similarly been used to construct CES-D scores in other major aging studies (e.g., HRS, ELSA, and KLoSA), this bias may extend beyond CHARLS and undermine the validity of depression findings across multiple international cohorts.

## Introduction

Inadequate handling of missing data can affect the precision and bias of model estimation and prediction, and compromise the credibility of study conclusions^1,2^. Existing research on missing data practices generally falls into two types. The first type of research are literature reviews that summarize how missing data are handled in different studies^3–7^. However, without being able to independently access and investigate the data used by each publication, such reviews cannot evaluate the appropriateness of the chosen missing data practices. The second type are methodological research that compare different missing data practices on a chosen dataset^8,9^. These studies inform readers how analytical results may change when using different missing data handling methods under various assumptions of how the missing values came about. Nonetheless, these results are based on hypothesized analyses for a specific dataset, and may not represent the range of methods that researchers actually use. Even though guidelines for handling missing data have been proposed for decades, it remains unclear whether applied researchers correctly adhere to these methodological principles in practice. We therefore use the China Health and Retirement Longitudinal Study (CHARLS) to address this deficiency. CHARLS is uniquely suited for this purpose due to the extensive use of its data, resulting in over 4,500 publications as of 2023^10^. This provides a substantial sample for evaluating real-world methodological practices, and ensures that our comparative findings are directly relevant to a large body of existing research.

We focus on the Center for Epidemiologic Studies Depression Scale (CES-D) as the outcome of interest since depression is highly prevalent among the middle-aged and elderly population and ranks second highest in China in terms of number of disability-adjusted life years^11,12^. It is also one of the extensively examined health outcomes in CHARLS: as of October 21, 2025, over 30% of the 4,879 publications using CHARLS data focused on depression. Our study will first review the literature and assess the methodological choices researchers have used to handle missing CES-D values in CHARLS. Second, based on a characterization of the CES-D’s missing values, we will compare the most frequently used methods on their depression prevalence estimates in the full sample and across key demographic dimensions such as sex and age. Ultimately, this work aims to provide objective evidence to enhance methodological standardization and robustness of conclusions in research on CES-D in CHARLS and other similar studies.

## Results

The H-CHARLS dataset exhibited a substantial proportion of missing data on the CES-D-10. Overall, 9% (*n* = 1,744) of the respondents did not answer any of the 10 items, and 19% (*n* = 3761) had missing data on at least one of the 10 items, implying that CCA on the CES-D-10 total score will also lose 19% of the participants. Missing rates for individual items were around 10%, with the items “I felt hopeful about the future” and “I had trouble keeping my mind on what I was doing” having the highest missing rates of 14% and 13%, respectively.

### Literature Review

CCA was the dominant approach to handle missing data, employed in 87% of the English articles and 92% of the Chinese articles. For English publications, higher CCA use was observed for lower impact journals, from 100% among D-tier papers to 63% among A-tier papers. In contrast, Chinese publications showed consistently high reliance on CCA (> 90%) regardless of the perceived prestige of their associated journals. CCA use remained stably high in English papers published between 2016–2020 and 2021–2025, but its use in Chinese papers showed a 10 percentage-point decrease to 90% during the second 5-year period. Multiple Imputation (primarily by chained equations) was the second most common method but only infrequently used (English papers: 8%; Chinese papers: 5%). Other methods such as full information maximum likelihood and random forest imputation were rarely used.

A critical finding is that 210 (96%) of the 219 sampled papers did not specify whether they used the original CHARLS dataset or the harmonized version, and the remaining 9 reported using the harmonized dataset. Of these 9 studies, 8 stated using CCA to handle missing values, implying an incongruence between their methodological description and the data actually used. This inconsistency is also probable among papers that did not specify the dataset being used since none of these studies mentioned recalculating the CES-D-10 total score using the original items, suggesting they could have used H-CHARLS dataset’s zero imputed CES-D-10 score by default. With respect to sensitivity analyses on handling missing data, only 6 papers (4 in English, 2 in Chinese) conducted them but all reported that alternative methods did not show substantive differences compared to their primary conclusions based on CCA.

#### Missing Mechanism for CES-D-10

Table 2 presents the subset of 28 covariates with the largest observed standardized differences (i.e., Cohen’s *h* > 2) in proportion of missing CES-D-10 data. For example, compared with the standardized difference of 0.1 between males and females, the standardized differences were 0.74 between the 75–95-year-old subgroup and the 45–54-year-old subgroup, 0.67 between those who use mobility devices and those who do not, and 0.58 between those with 1–2 living children and those with none. These values represent medium-to-large effect sizes and absolute differences of 25 to 32 percentage points, indicating that CES-D-10 items are more likely to be missing among individuals of older age, poorer health status, and weaker social support. Together with the other covariates in Table 2, these results indicate that missing values in the CES-D-10 items are strongly dependent on these variables. Consequently, the CES-D-10 items cannot be missing completely at random, thus invalidating a key assumption for using CCA.

#### Impact of Missing Data Handling Methods on Depression

**Figure 1:**
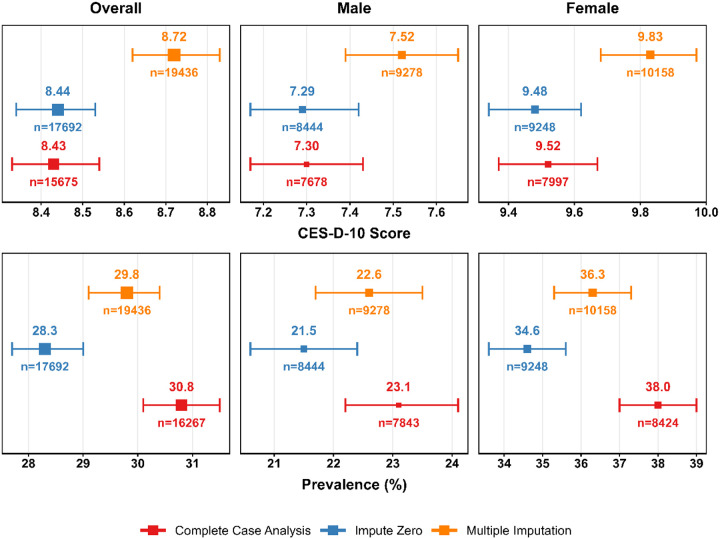
Forest Plot of CES-D-10 Total Score and Depression Prevalence by Missing Data Handling Method

Compared with MI, both IZ and CCA yielded similar but underestimated CES-D-10 mean scores. The total sample sizes for IZ and CCA, however, differed by > 2000, a discrepancy that can affect their apparent comparability. Estimated depression prevalence was 30% under MI, 28% under IZ (−2 percentage points), and 31% under CCA (+ 1 percentage point). Sex differences also followed this pattern: 13% for IZ, 14% for MI, and 15% for CCA (Fig. 1).

**Figure 2:**
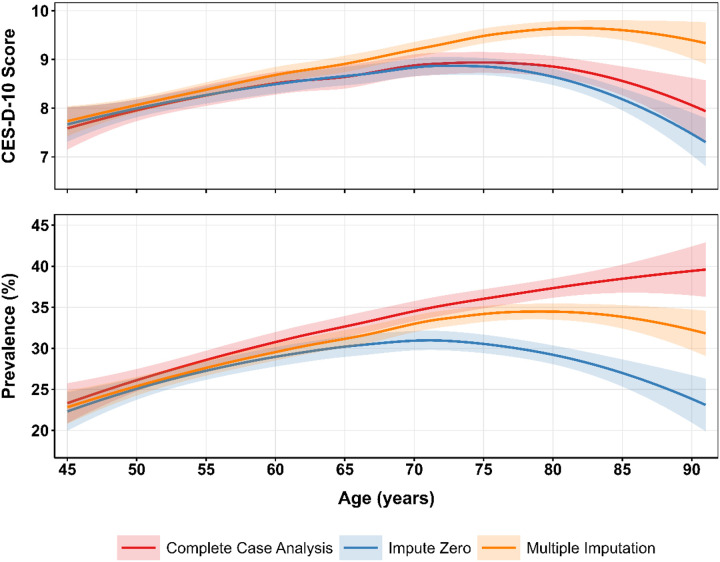
LOWESS Curves of CES-D-10 Total Score and Depression Prevalence by Missing Data Handling Method

Figure 2 indicates that all three methods produced similar upward age trajectories until about age 70. Afterwards, the trends diverged by outcome and method. For the CES-D-10 score, the IZ and CCA curves flattened after age 70 and then declined, with the IZ curve having a slightly steeper descent, whereas the MI curve continued to increase until age 80 before declining moderately. For depression prevalence, the MI curve increased slowly and slightly after age 70 and only began declining around age 85. In contrast, the IZ curve fell sharply after age 70, substantially understating risk, whereas the CCA curve continued to rise, leading to overestimation.

**Figure 3:**
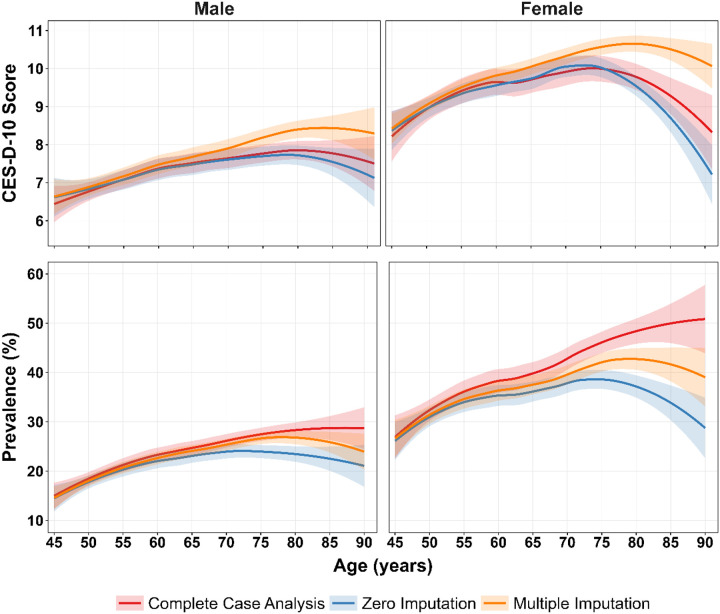
Sex-specific LOWESS Curves of CES-D-10 Total Score and Depression Prevalence by Missing Data Handling Method

A sex-stratified analysis of the age trends revealed that differences between the methods in the overall sample were more pronounced in women than in men since the age trajectories were less separated among men even at older ages (Fig. 3). Among adults aged 75 + years, the spread in CES-D-10 scores and prevalence was considerably wider in women. For example, among men, the IZ CES-D-10 score and prevalence declined modestly from 7.7 and 24% at age 75 to 7.2 and 21% at age 90, whereas among women these values dropped from 10.0 and 39% to 7.3 and 29%, respectively. Among women aged 75 + years, marked differences in depression risks were observed between the methods: prevalences were 36% (IZ), 42% (MI), and 47% (CCA), and female-male differences were 13% (IZ), 15% (MI), and 19% (CCA).

## Discussion

Depressive symptoms are embedded in several composite health indicators, including frailty indices and measures of health-related quality of life. Consequently, suboptimal handling of missing CES-D data has implications that extend beyond studies of depression itself, potentially compromising the validity of a wide range of health research based on CHARLS. Our empirical comparisons between the three most commonly used missing data handling methods for the CES-D in CHARLS reveal non-trivial differences in estimates of core constructs like mean symptom score and prevalence. These disparities are especially pronounced among late elderly women, suggesting that methodological choices may disproportionately affect inference about vulnerable subpopulations. Notably, different handling methods produced markedly divergent depression trajectories in women aged 70+. Such variation may explain why some studies reported an inverse relationship between depression prevalence and age among adults over 60^13,14^, while others showed steadily increasing depression symptoms among aging women^15^.

We uncovered a systemic disconnect between theory and practice in terms of handling missing CES-D-10 items in CHARLS since nearly 90% of our surveyed studies employed CCA as the primary or sole method despite the pitfalls of CCA being known for decades. Unless the data are missing completely at random or missing under certain restrictive circumstances, CCA not only leads to precision loss but also biased estimates^1,16,17^. However, none of these studies examined whether the missing mechanism was dependent on measured covariates, perhaps naively assuming that the data would be missing completely at random. Beyond theoretical limitations, studies applying CCA may control for different sets of covariates according to their specific research objectives, thereby ending up with different sample sizes for analysis. This heterogeneity directly contributes to significant variation in reported findings, compromising comparability across studies^18^. For example, even with identical depression cutoffs and similar age groupings, different studies have reported sample sizes ranging from 5,207 to 14,148 and prevalences varying from 36% to 45%^19–21^. CCA is therefore in practice a highly contextualized process influenced by the researcher’s subjective variable selection strategies.

A central finding of this study reveals that treating missing CES-D-10 items as equivalent to zero in the H-CHARLS dataset constitutes an important yet unrecognized source of systematic bias that can substantially distort depression estimates and trajectories, especially among late elderly females. This methodological caveat appears to be largely overlooked in existing research as 96% of the studies in our review did not specify whether they used the harmonized or the original CHARLS datasets. More importantly, among the few studies that explicitly stated using H-CHARLS data, all reported using CCA or full information maximum likelihood to handle missing values, suggesting that the authors were either unaware of or ignored how missing CES-D-10 items were processed in the H-CHARLS dataset. This inconsistency likely extends to other studies that did not specify their data source, raising broader concerns about the transparency and credibility of their conclusions. Furthermore, summary measures of temporal orientation in the cognitive module, Activities of Daily Living (ADL), Instrumental Activities of Daily Living (IADL), mobility, and the Parental Warmth scale were also constructed using the IZ method in the H-CHARLS dataset. If this is the Gateway to Global Aging Data (GGAD) team’s methodological practice in handling missing items in health and psychological scales, then the bias due to the IZ method will affect not just findings from CHARLS but also those from other international aging studies that they managed.

The above comments merit several recommendations. First, the GGAD team should forego using IZ when constructing summary scales for the harmonized datasets since IZ may lead to serious underestimation. Although these pre-calculated scales were intended as a convenience for users, the fact that some users misinterpreted how the CES-D-10 was generated suggests that it may be preferable for individuals to compute their own summary scores. Second, publications need to explicitly declare the source and version of the CHARLS dataset being used. Third, researchers should systematically evaluate the missing-data mechanism when analyzing variables such as the CES-D-10 that contain > 10% missing values – for example, by examining how the proportion of missing data varies across covariates, as illustrated in Table 2 – rather than naively assuming the validity of CCA. Sensitivity analyses can be informative, but only when the methods are transparently documented. For instance, multiple imputation must specify the predictors included in the imputation model to ensure reproducibility. Finally, reviewers and readers should scrutinize methodological details, especially data sources and missing data handling. When interpreting studies of depression in CHARLS, readers should expect authors to carefully justify their approach to processing missing values as a critical step for evaluating the validity and generalizability of the study’s conclusions.

## Methods

### 1. Sampling Design and Data Source

CHARLS is a nationally representative household survey that collects multidimensional information related to aging on Chinese residents aged 45 years and above. It employed a multistage (county-, neighborhood-, household-, and respondent-level) proportional to population size random sampling scheme that is stratified by the per capita GDP of the urban districts/rural counties. The baseline survey was initiated in 2011 with a final sample consisting of 10,200 + households from 450 villages/communities selected from 150 counties in 28 provinces. The baseline sample’s age structure and marital status were quite similar to those of the 2010 China Census^22^. Follow-up surveys were conducted every two to three years, and further details are described by Zhao et al. (2020). CHARLS had been approved by the Biomedical Ethics Review Committee of Peking University.

CHARLS was designed to ensure comparability with other international aging studies such as the U.S. Health and Retirement Survey, English Longitudinal Study of Aging, and the Survey of Health, Aging and Retirement in Europe. The University of Southern California Gateway to Global Aging Data team likewise constructed a harmonized CHARLS (H-CHARLS) dataset that incorporated the baseline and first three follow-up surveys to assist such cross-country comparisons. Our analysis on the effects of missing values on CES-D will focus on the most recent 2018 survey in the H-CHARLS dataset. To minimize the influence of extreme ages on modeling, the analytical sample was restricted to respondents aged 45 to 95 years, resulting in a maximum total sample size of 19,436 participants.

### 2. Measures

#### Depression

Depressive symptoms were assessed in CHARLS using the 10-item CES-D (CES-D-10), which measures the frequency of depressive affect and somatic symptoms experienced during the preceding week. Responses were recorded on a 4-point Likert scale: 0 = rarely or none of the time (< 1 day), 1 = some or a little of the time (1 ~ 2 days), 2 = occasionally or a moderate amount of the time (3 ~ 4 days), and 3 = most or all of the time (5 ~ 7 days). Total scores ranged from 0 to 30, with higher scores indicating more severe depressive symptoms. The CES-D-10 has demonstrated good validity and reliability in Chinese middle-aged and older populations^23^, and we adopted a cut-off score of ≥ 12 to designate individuals with clinically significant depressive symptoms^24^

#### Predictors

Based on a literature review on late-life depression, we identified potential predictors spanning demographics, socioeconomic status, health conditions, functional limitations, lifestyle, and subjective well-being, and selected 42 self-reported variables collected in the 2018 questionnaire for our missing data analysis.

### 3. Analysis

#### 3.1 Literature Review

For Chinese-language publications, we searched the China National Knowledge Infrastructure database on September 9, 2025 using the keywords: (‘CHARLS’ OR ‘中国健康与养老追踪调查’) AND (‘抑郁’ OR ‘抑郁症’ OR ‘抑郁症状’). This search retrieved 105 journal articles and 121 dissertations, and we restricted our analysis to the 105 articles. One article did not provide any information on the CES-D, leaving 104 articles for final inclusion.

For English-language publications, we searched PubMed on July 7, 2025 and yielded 1,068 publications using the keywords: (“CHARLS” OR “China Health and Retirement Longitudinal Study”) AND (“CES-D” OR “Center for Epidemiologic Studies Depression Scale” OR “depressive symptoms” OR depression). We separated the publications into four tiers based on their journal’s impact metrics: 8 A-tier papers with impact factor (IF) > 10, 264 B-tier papers with IF 5–10, 612 C-tier papers with IF < 5, and 184 D-tier papers and conference proceedings not included in the Science Citation Index. To obtain a similar sample size as the number of Chinese publications and to ensure representation across the four tiers of academic impact, we selected all 8 of the A-tier papers and randomly sampled 15% (38) B-tier papers, 10% (60) C-tier papers, and 5% (9) D-tier papers, resulting in a final sample of 115 English articles.

Each publication was independently reviewed by two researchers to decipher the statistical method used for handling missing CES-D values. In instances of disagreement, adjudication was provided by a third researcher.

#### 3.2 Missing Data Analysis

##### Multiple Imputation

To assess the missingness mechanism for CES-D-10, we first compared the distribution for each of the 42 predictors between respondents with and without any missing CES-D-10 items using Cohen's *h*, retaining those with non-small effect sizes or |*h*|≥0.2. Next, we applied least absolute shrinkage and selection operator regression on the missing indicator variable using all the predictors, and identified the variables with non-zero regression coefficients. The union of the variables from these two strategies were included in the missing values imputation model. The final imputation model included the ten CES-D-10 items and 28 covariates: sex (female/male), age group (45–54, 55–64, 65–74, 75–95 years), marital status (married, widowed, never married or divorced), education level (< lower secondary, upper secondary and vocational training, tertiary), household registration type (agricultural, non-agricultural, unified), type of residential address (family housing, nursing home or hospital), residential location (urban/rural), currently employed (no/yes), pension plan (no/yes), number of living children (0, 1–2, > 2), satisfaction with children (no/yes), public health insurance enrollment (no/yes), social activity participation (no/yes), frequent alcohol consumption in past year (no/yes), life satisfaction (satisfied/dissatisfied), health satisfaction (satisfied/dissatisfied), self-rated health (good, fair, poor), bodily pain (no/yes), sleep duration (< 6 hrs., 6–8 hrs., > 8 hrs.), physical disability (no/yes), Instrumental Activities of Daily Living (IADL) disability (no/yes), mobility device use (no/yes), dyslipidemia (no/yes), heart disease (no/yes), lung disease (no/yes), hip fracture (no/yes), stroke (no/yes), and kidney disease (no/yes).

Multiple imputation (MI) was performed via chained equations using the mice package (version 3.17.0) in R software (version 4.4.1). Five imputed values were generated for each missing value, and results were pooled according to Rubin's rule. The maximum number of iterations within a single imputation was set to 10. Proportional odds regression was used to impute missing values for the CES-D-10 items and other ordinal variables, while multinomial logistic regression was used to impute nominal variables.

##### Impute Zero

In the H-CHARLS dataset, a CES-D-10 total score was constructed as the sum of the available responses from the 10 items, and was considered missing only if all 10 items had missing values, resulting in a sample size of 17692. This approach is mathematically equivalent to imputing missing items with a score of zero, thus we label it as the impute zero (IZ) method.

#### 3.3 Statistical Analysis

We evaluated three commonly used methods (complete case analysis (CCA), impute zero, and multiple imputation) for handling missing CES-D-10 items, and compared their corresponding mean CES-D-10 total score and prevalence of depression. For CCA, we retained 15675 individuals with complete data for all 10 items when calculating the total CES-D-10 score, but included additional individuals (*n* = 16267) whose total score from the available items was already ≥ 12 when calculating the prevalence as they would be classified as depressed regardless of the missing values from the other items. Locally weighted scatterplot smoothing (LOWESS) was used to visualize age trends. To ensure stable trend estimates, age groups with less than 30 observations were merged with adjacent groups, and the mean age of the groups was used as the x-coordinate.

## Figures and Tables

**Table 1:** Characteristics of Manuscripts According to How They Handled Missing CES-D-10 Data in CHARLS

Database	Method	Total N(%)	Publication Period		Journal Impact Quality			
			2016–2020 N(%)	2021–2025 N(%)	IF > 10 N(%)	IF 5–10 N(%)	IF < 5 N(%)	Non-SCI N(%)
PubMed (N = 115)	CCA	100(86.96)	11(84.62)	89(87.25)	5(62.50)	32(84.21)	54(90.00)	9(100)
	MICE	9(7.82)	1(7.69)	8(7.84)	2(25.00)	2(5.26)	5(8.33)	0
	FIML	5(4.35)	1(7.69)	4(3.93)	0	4(10.53)	1(1.67)	0
	EM	1(0.87)	0	1(0.98)	1(12.5)	0	0	0
					Double Core N(%)	PKU Core N(%)	Other N(%)	
CNKI (N = 104)	CCA	96(92.31)	27(100%)	69(89.61)	16(94.12)	41(91.11)	39(92.86)	
	MICE	5(4.81)	0	5(6.49)	1(5.88)	3(6.67)	1(2.38)	
	RF	2(1.92)	0	2(2.60)	0	1(2.22)	1(2.38)	
	IZ	1(0.96)	0	1(1.30)	0	0	1(2.38)	

1. CNKI, China National Knowledge Infrastructure; IF, journal impact factor; SCI, science citation index.

2. “Double Core” indicates simultaneous indexing in both the Peking University Core (PKU) and Chinese Social Sciences Citation Index (CSSCI), representing the most prestigious journals in China.

3. CCA, complete case analysis; IZ, impute zero; MICE, multiple imputation by chained equations; FIML, full information maximum likelihood; EM, expectation-maximization algorithm; RF, random forest imputation.

**Table 2:** Frequency and Percentage of Missing CES-D Data by Covariate Level

Age Group	No Missing CES-D Items (N = 15675)	Any Missing CES-D Items (N = 3761)	Cohen's *h*
		
45–54	5014 (87.29%)	730 (12.71%)	—
55–64	5367 (84.97%)	949 (15.03%)	0.07
65–74	3991 (79.61%)	1022(20.39%)	0.21
75–95	1303 (55.14%)	1060 (44.86%)	0.74
**Mobility Device Use**			
No	14935 (83.05%)	3048 (16.95%)	—
Yes	720 (52.79%)	644 (47.21%)	0.67
**Number of Living Children**			
1–2	9492 (85.89%)	1559 (14.11%)	—
>2	6064 (74.05%)	2125 (25.95%)	0.30
0	119 (60.71%)	77 (39.29%)	0.58
**IADL Disability**			
No	12116 (85.87%)	1993 (14.13%)	—
Yes	3539 (67.56%)	1699 (32.44%)	0.44
**Marital Status**			
Married	13730 (82.97%)	2818 (17.03%)	—
Never married or divorced	315 (77.21%)	93 (22.79%)	0.14
Widowed	1630 (65.73%)	850 (34.27%)	0.40
**Currently Working**			
No	5159 (73.25%)	1884 (26.75%)	—
Yes	10497 (85.34%)	1803 (14.66%)	0.30
**Self-reported Health Status**			
Good	4048 (90.62%)	419 (9.38%)	—
Fair	3828 (81.27%)	882 (18.73%)	0.27
Poor	7781 (88.59%)	1002 (11.41%)	0.07
**Stroke**			
No	14671 (81.50%)	3331 (18.50%)	—
Yes	999 (70.65%)	415 (29.35%)	0.26
**Physical Disabilities**			
No	14205 (81.76%)	3170 (18.24%)	—
Yes	399 (70.87%)	164 (29.13%)	0.26
**Satisfaction with Children**			
Yes	14924 (88.38%)	1963 (11.62%)	—
No	628 (79.29%)	164 (20.71%)	0.25
**Health Insurance**			
Yes	15265 (81.13%)	3550 (18.87%)	—
No	409 (70.76%)	169 (29.24%)	0.24
**Fractured Hip**			
No	15319 (81.13%)	3564 (18.87%)	—
Yes	156 (71.23%)	63 (28.77%)	0.23
**Life Satisfaction**			
Satisfied	13993 (88.74%)	1775 (11.26%)	—
Dissatisfied	1682 (81.26%)	388 (18.74%)	0.21
**Sex**			
Male	7678 (82.75%)	1600 (17.25%)	—
Female	7997 (78.73%)	2161 (21.27%)	0.10

## Data Availability

The datasets generated and/or analyzed during the current study are available in the China Health and Retirement Longitudinal Study repository, https://charls.charlsdata.com/.
